# Summary of the best evidence for non-pharmacological management of dysphagia in Parkinson’s disease patients

**DOI:** 10.3389/fneur.2026.1810325

**Published:** 2026-06-15

**Authors:** Jun Cao, Zhen Ning, Liru Hao, Yanqun Zhang, Yichun Zhang, Lidan Pang, Xiuping Ye, Song Zhou

**Affiliations:** 1 Department of Neurology, The Fifth Affiliated Hospital of Sun Yat-sen University, Zhuhai, Guangdong, China; 2 Department of Outpatient, The Fifth Affiliated Hospital of Sun Yat-sen University, Zhuhai, Guangdong, China; 3 School of Nursing, Guangzhou Medical University, Guangzhou, Guangdong, China

**Keywords:** drooling, dysphagia, evidence summary, evidence-based nursing, management, Parkinson’s disease

## Abstract

**Objective:**

This study systematically synthesizes the best available evidence for the non-pharmacological management of swallowing disorders in Parkinson’s disease, establishing an actionable framework to standardize clinical implementation and optimize multidisciplinary care pathways.

**Methods:**

Based on the “6S” evidence resource model, a systematic search was conducted for all evidence on dysphagia management in Parkinson’s disease, including guidelines, expert consensuses, clinical decision-making tools, and systematic reviews. The search period spanned from the inception of the database up to December 31, 2024. Three researchers independently screened and evaluated the literature, and subsequently, two researchers extracted and summarized the evidence in accordance with the JBI grading of evidence and recommendation system.

**Results:**

A total of 18 pieces of literature were included, encompassing 2 guidelines, 4 expert consensuses, 11 systematic reviews, and 1 clinical decision-making document. The evidence covered 7 categories, with a total of 53 best evidence items identified, including screening, assessment, rehabilitation, nutritional management, airway and complication management, and outcome evaluation.

**Conclusion:**

This study comprehensively and scientifically summarizes the best evidence for the non-pharmacological management of swallowing disorders in patients with Parkinson’s disease. Users of this evidence should consider specific clinical contexts during the translation of evidence and select appropriate evidence-based interventions to develop individualized and localized management plans.

## Introduction

Parkinson’s disease (PD), also known as paralysis agitans, is the second most common neurodegenerative disease following Alzheimer’s (1). It is primarily characterized by clinical motor symptoms such as resting tremor, bradykinesia, unstable gait, and muscle rigidity, as well as non-motor symptoms including autonomic nervous system dysfunction, sleep disorders, cognitive impairment, and abnormal mental behaviors (1). Approximately 36.9 to 80% of PD patients experience dysphagia (2–4). Among them, about 10.87 to 34.78% of the patients develop aspiration pneumonia (5). The prevalence may be influenced by various factors. These include assessment methods, such as clinical screening and instrumental examinations. Definitions of dysphagia also contribute, for example, subjective complaints versus objective functional indicators. Nevertheless, overall, the aforementioned prevalence-related symptoms have increased the burden on caregivers but also raises the hospitalization and mortality rates of patients, severely affecting their mental health, quality of life, and survival rate (6). Some studies have indicated (7, 8) that early screening and assessment are crucial for the management of dysphagia in PD patients. Rehabilitation training and compensatory strategies can effectively improve the patients’ swallowing function, reduce the risk of aspiration and incidence of pneumonia (7, 8). Currently, studies on managing dysphagia in patients with Parkinson’s disease (PD) are scattered and vary in their levels of evidence. Consequently, this study conducted a comprehensive literature search on non-pharmacological approaches to dysphagia in PD. It summarized the highest quality evidence using evidence-based nursing methodologies, with the aim of providing a foundation for clinical practice in managing dysphagia in PD patients.

## Methods

### Problem establishment

This study employed the question development tool in conjunction with the PIPOST model (9). ① “P (population)” denotes the target population for the application of evidence, specifically patients with Parkinson’s disease (PD). ② “I (intervention)” refers to the intervention measures, particularly the management strategies for dysphagia. ③ “P (professional)” signifies the professionals engaged in the application of evidence, including nurses, physicians, physical therapists, and others. ④ “O (outcome)” pertains to the outcome indicators, which include primary outcomes such as the incidence of aspiration or choking and the risk of pneumonia, as well as secondary outcomes like swallowing safety, food residue, and other risk factors related to aspiration or choking. ⑤ “S (setting)” indicates the environments where the evidence is applied, encompassing neurology wards and home settings. ⑥ “T (type of evidence)” refers to the categories of evidence resources, including guidelines, expert consensus, systematic reviews, and clinical decision-making.

Using this tool, we defined the specific clinical question for this evidence summary. This step ensured that the subsequent search strategy was both comprehensive and specific.

“Clinical decision” refers to resources that directly guide clinical practice. These include evidence-based decision-support tools (e.g., UpToDate), topic summaries, and standardized recommendations from professional societies. Ultimately, we included one article by Evatt et al. (10) as a “clinical decision” resource. This article integrates evidence to guide specific clinical assessment decisions.

The study has been enrolled in PROSPERO under the registration number of CRD420251030197. The entire document is consistent with the information provided during registration and has not undergone significant changes.

### Evidence retrieval

**Figure 1:**
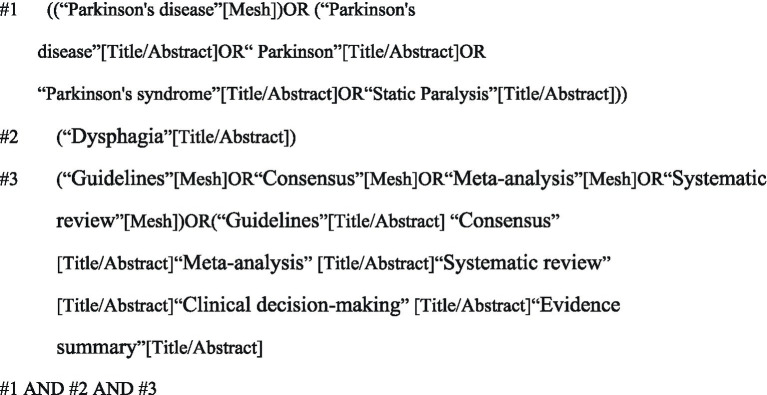
Searching strategy of PubMed.

According to the “6S” evidence model (11), A comprehensive, top-down search was conducted across the following evidence-based resources: UpToDate, BMJ Best Practice, the Joanna Briggs Institute (JBI) EBP Database (Australia), the Guidelines International Network (GIN), the AHRQ National Guideline Clearinghouse (US), the UK National Institute for Health and Care Excellence (NICE), the Registered Nurses’ Association of Ontario (RNAO) (Canada), the European Parkinson’s Disease Association, the China Guideline Clearinghouse, and Medlive. Supplementary searches were conducted in comprehensive databases, including PubMed, the Cochrane Library, CINAHL, Embase, Web of Science, SinoMed, CNKI, Wanfang Database, VIP Database, and Chinese Medical Association Journals. When searching for clinical decision-making resources, recommended practice databases, clinical guideline repositories, and professional society websites, the following search terms were utilized: “Parkinson’s disease,” “Parkinson,” “Parkinson’s syndrome,” “Static Paralysis,” “Dysphagia.” The comprehensive database includes additional search terms such as “Guidelines,” “Consensus,” “Meta-analysis,” “Systematic review,” “Clinical decision-making,” and “Evidence summary.” A combination of subject headings and free-text terms was used. The search period spanned from the inception of the database to December 31, 2024. The search was limited to articles published in Chinese and English. The search strategy was adapted to the requirements of each database. For instance, PubMed’s detailed search strategy is illustrated in Figure 1.

### Inclusion and exclusion criteria for literature selection

Inclusion Criteria: ① Population: Studies involving patients with Parkinson’s disease (PD). Patients with secondary parkinsonism, parkinsonian-plus syndromes (e.g., multiple system atrophy [MSA] and progressive supranuclear palsy [PSP]), and other atypical parkinsonian disorders were excluded to ensure homogeneity of the study population. ② Content: Research focusing on the non-pharmacological management of dysphagia in PD patients. This encompassed, but was not limited to, screening and assessment of swallowing function (e.g., clinical scales and instrumental examinations), monitoring of aspiration and choking risks, rehabilitation therapies (including behavioral therapy, swallowing muscle exercises, biofeedback, and respiratory-swallow coordination training), compensatory strategies (e.g., postural adjustments and food texture modification), nutritional management, airway management, and prevention and management of associated complications. Studies exclusively employing pharmacological treatments (e.g., levodopa and dopamine agonists) or surgical interventions (e.g., deep brain stimulation) as primary therapeutic strategies were excluded. ③ Study Types: Clinical guidelines, expert consensus statements, systematic reviews, clinical decision-making studies. ④ Language: Publications in English or Chinese only.

Exclusion Criteria: ① Studies with incomplete data, unavailable full-text articles, or duplicate publications. ② Quality: Studies that, after assessment using the standardized tools described in the “Literature quality assessment” section below, were rated as low quality and thus excluded. Specifically: a. For guidelines, those with an overall AGREE II rating of “C” (low quality) were excluded. b. For systematic reviews, expert consensuses, and evidence summaries (clinical decision-making), studies for which the majority of critical appraisal items were assessed as “no” (indicating failure to meet methodological standards) were excluded.

### Literature quality assessment

#### Guidelines

The Appraisal of Guidelines for Research and Evaluation II (AGREE II) (12) was utilized. This tool consists of six domains, each containing 23 items. Each item is rated on a scale from 1 to 7. Domain scores are calculated by adding the scores of all items within each domain and then standardizing these totals. Typically, a guideline is classified as Grade A if the standardized percentage score for all six domains exceeds 60%; it is classified as Grade B if at least three domains have scores ranging from 30 to 60%; and it is classified as Grade C if at least three domains have scores below 30%.

#### Systematic reviews

We used the Systematic Review Quality Assessment Tool developed by the Joanna Briggs Institute (JBI) Centre for Evidence-Based Healthcare in Australia (2016 version) (13). This tool consists of 11 items, which are assessed using the options “yes,” “no,” “unclear,” and “not applicable.”

#### Expert consensus

We used the Critical Appraisal Tool for Expert Consensus, developed by the JBI Centre for Evidence-Based Healthcare in Australia (2016 version) (14). This tool consists of six items, and the appraiser must render a judgment of “yes,” “no,” “unclear,” or “not applicable” for each one.

The expert consensus literature included in this study was developed following structured consensus procedures. The primary method employed is the modified Delphi method. The core process involves: forming a multidisciplinary expert panel, drafting preliminary recommendations based on literature, conducting multiple rounds of anonymous questionnaire surveys and statistical analysis (with a typical consensus threshold, e.g., agreement rate ≥75%), and holding a consensus meeting to finalize the recommendations. Participants are authoritative experts from multiple relevant fields such as neurology, rehabilitation medicine, speech-language pathology, and nursing. Specific details regarding the expert panel composition are available in the original publications (15–18).

#### Clinical decision-making

Clinical decision-making falls under the category of topic-specific evidence summaries within the “6S” evidence model (11). The quality of evidence summaries was evaluated using a specific quality assessment tool designed for evidence summaries (19), which includes 10 items, each of which is evaluated as “yes,” “partially yes,” or “no.”

The literature was independently assessed by three researchers with systematic training in evidence-based nursing. In instances of conflicting opinions, a fourth researcher—an expert in evidence-based nursing—along with other research team members, engaged in discussions to achieve a consensus.

The quality assessment for each included piece of literature was conducted independently by three researchers (JC, ZN, and LH) who have undergone systematic training in evidence-based nursing. In instances of conflicting opinions, a fourth researcher—an expert in evidence-based nursing—along with other research team members, engaged in discussions to achieve a consensus.

### Evidence extraction and synthesis

The evidence was synthesized according to the following principles: ① Consistent Recommendations: Recommendations with identical content were combined. ② Complementary Recommendations: Recommendations featuring complementary content were merged into a comprehensive recommendation. ③ Conflicting Recommendations: Prioritizes high-quality evidence, followed by recently published literature, and then literature from authoritative journals. ④ Independent Content Items: Items with independent content were retained in their original wording.

The JBI Evidence Pre-Grading and Evidence Recommendation Level System (2014) was used to pre-grade the evidence (20). The evidence levels are categorized into Grades 1 to 5. The grading criteria are as follows: Grade 1 corresponds to randomized controlled trials and other experimental studies, Grade 2 to quasi-experimental studies, Grade 3 to observational-analytical studies, Grade 4 to observational-descriptive studies, and Grade 5 to expert opinions and fundamental research. Grade 1 represents the highest level of evidence, while Grade 5 represents the lowest level.

Under the guidance of the JBI FAME framework, the strength of the recommendations was determined as Grade A (strong recommendation) or Grade B (weak recommendation) following group discussion, based on the evidence’s validity, applicability, appropriateness, and clinical significance (20). The extracted evidence was reviewed during a consensus meeting, and the corresponding strength of recommendation was determined through discussion and voting.

During the data extraction phase, two researchers independently extracted the bibliographic information, evidence content, and methodological details using standardized forms, and then, in collaboration with other members of the research team, they discussed, translated, proofread, and integrated it. For English-language evidence, designated personnel performed independent translation followed by cross-verification. Evidence synthesis involved merging consistent and complementary contents, with conflicts resolved according to the prioritization hierarchy of “evidence quality—publication recency—source authority.” During evidence synthesis, we noted that different sources (e.g., guidelines, systematic reviews) may cite the same original studies. To minimize potential bias from this overlap, the following strategies were adopted: during extraction, shared original studies were annotated; during integration, recommendations based on identical core studies were merged into a single entry with all supporting literature cited; and during evidence grading, the highest design level of the core original studies was prioritized over merely accumulating the number of references. This approach ensured the rigor of the conclusions. Additionally, “independent content items” were explicitly defined as single, complete recommendation entries that could not be merged with other recommendations.

### Strengths and limitations of the study methodology

This study employed the “6S” model and PIPOST framework for systematic evidence retrieval and synthesis (11), with methodological strengths including standardized procedures, comprehensive evidence chaining, and rigorous quality appraisal, further enhanced by the FAME framework to strengthen clinical translation orientation (20). Nevertheless, conclusions are constrained by the currency and methodological quality of included studies, potential language bias may exist, and the generalizability of certain interventions across diverse healthcare settings warrants further validation.

## Results

### Literature search results

**Figure 2:**
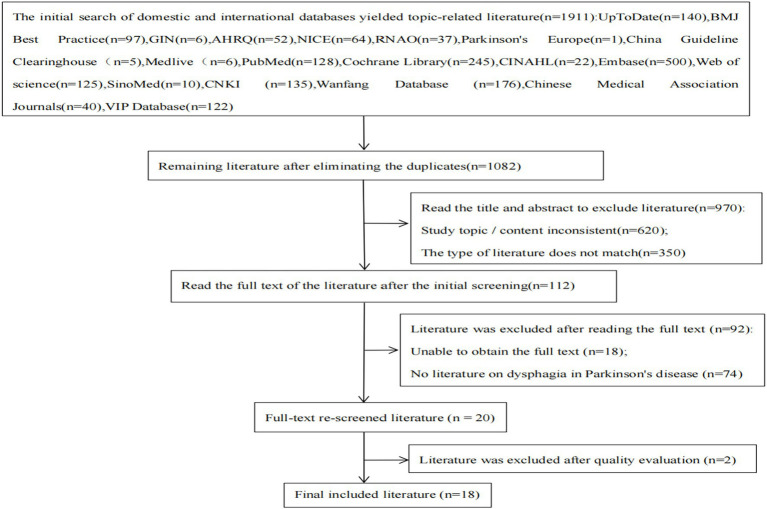
Flow chart of literature screening.

A total of 1,911 articles were retrieved. Following deduplication, preliminary screening, detailed screening, and quality assessment, 18 articles were ultimately included. Among them, there were 2 guidelines (21, 22), 11 systematic reviews (23–33), 4 expert consensuses (15–18), and 1 clinical decision (10). The literature search process is depicted in Figure 2, and the fundamental characteristics of the included articles are outlined in Table 1.

**Table 1:** Basic characteristics of included literature (*n* = 18).

Included literature	Publication year	Country/Region	Source of literature	Literature type	Research focus
Dziewas et al. (21)	2021	Germany	PubMed(Neurological Research and Practice)	Clinical Guideline	Diagnosis and treatment of neurogenic dysphagia
Burgos et al. (22)	2018	Spain	PubMed (Clin Nutr)	Clinical Guideline	Nutritional management in neurogenic dysphagia
Siyuan Gong et al. (23)	2023	China	CNKI (Journal of Nursing)	Systematic Review	Content, advantages, limitations, and recommendations of dysphagia screening tools in Parkinson’s disease
Yiwen Liu et al. (24)	2023	China	CNKI (China Medicine and Pharmacy)	Systematic Review	Efficacy and safety of acupuncture therapy for dysphagia in Parkinson’s disease
Wu et al. (25)	2023	China	PubMed (Frontiers in Neurology)	Systematic Review	Therapeutic effects of acupuncture on dysphagia in Parkinson’s disease
Winiker et al. (26)	2023	Switzerland	PubMed (Lang Commun Disord)	Systematic Review	Intervention methods and evaluation of dysphagia in Parkinson’s disease
Javorszky et al. (27)	2024	Austria	PubMed (BMC Geriatr)	Systematic Review	Screening and intervention for dysphagia and nutritional disorders
Cheng et al. (28)	2023	UK	PubMed (Neurogastroenterol Motil)	Systematic Review	Treatment of dysphagia in Parkinson’s disease
Gandhi et al. (29)	2022	Canada	PubMed (Am J Speech Lang Pathol)	Systematic Review	Interventions for dysphagia in Parkinson’s disease
Kim et al. (30)	2023	South Korea	PubMed (Int J Speech Lang Pathol)	Systematic Review	Behavioral swallowing therapy in Parkinson’s disease
Thijs et al. (31)	2023	USA	PubMed (Laryngoscope Investig Otolaryngol)	Systematic Review	Assessment of laryngeal symptoms in Parkinson’s disease
Atkin et al. (32)	2024	UK	PubMed (Eur Geriatr Med)	Systematic Review	Impact of thickeners on drug efficacy in dysphagia treatment
Van Hooren et al. (33)	2014	Netherlands	PubMed (Dysphagia)	Systematic Review	Treatment and rehabilitation of dysphagia in Parkinson’s disease
Chinese PD Consensus Group et al. (15)	2020	China	CMA (Chinese Medical Journal)	Expert Consensus	Management of non-motor symptoms in Parkinson’s disease
Chinese Dysphagia Consensus Group et al. (16)	2024	China	Wan Fang database (Physical Medicine and Rehabilitation)	Expert Consensus	Screening, assessment, rehabilitation, and nutritional management of dysphagia in Parkinson’s disease
Cosentino et al. (17)	2022	Italy	PubMed (Neurol)	Expert Consensus	Screening, diagnosis, impact, and prognosis of dysphagia in Parkinson’s disease
Schindler et al. (18)	2021	Italy	PubMed (Neurol Sci)	Expert Consensus	Treatment protocols, training, and nutritional strategies for dysphagia in Parkinson’s disease
Evatt et al. (10)	2009	USA	PubMed (Mov Disord)	Clinical Decision-Making	Evaluation scales for autonomic dysfunction (sialorrhea, dysphagia, constipation) in Parkinson’s disease and their clinical implications

### Literature quality evaluation results

#### Guidelines

The two included guidelines (21, 22) were both rated as high quality (“Grade A”) across all AGREE II domains, as detailed in Table 2.

**Table 2:** Quality evaluation results of guidelines (*n* = 2).

Included guideline	Standardized percentage per domain (%)						≥60% field number	≥30% field number	Quality rating
	Scope and purpose	Stakeholder involvement	Rigor of development	Clarity of presentation	Applicability of the guide	Independence of the guide’s compilation			
Dziewas et al. (21)	100.00	85.19	88.19	96.30	86.11	100.00	6	6	A
Burgos et al. (22)	100.00	75.93	84.03	100.00	100.00	100.00	6	6	A

#### Systematic review

Of the 11 included systematic reviews (23–33), the majority were assessed as high quality. Specific methodological limitations identified for individual reviews are summarized in Table 3. All were deemed to meet the minimum quality threshold for inclusion.

**Table 3:** Quality evaluation results of the system reviews (*n* = 11).

Incorporate into systematic review	①	②	③	④	⑤	⑥	⑦	⑧	⑨	⑩	⑪
Siyuan Gong et al. (23)	Yes	Yes	Yes	Yes	No	Yes	Yes	Yes	No	Yes	Yes
Yiwen Liu et al. (24)	Yes	Yes	Yes	Yes	Yes	Yes	Yes	Yes	Yes	Yes	Yes
Wu et al. (25)	Yes	Yes	Yes	Yes	Yes	Yes	Yes	Yes	Yes	Yes	Yes
Winiker et al. (26)	Yes	Yes	Yes	Yes	Yes	Yes	Yes	Yes	Unclear	Yes	Yes
Javorszky et al. (27)	Yes	Yes	Yes	Yes	Unclear	Yes	Yes	Yes	Unclear	Yes	Yes
Cheng et al. (28)	Yes	Yes	Yes	Yes	Yes	Yes	Yes	Yes	Yes	Yes	Yes
Gandhi et al. (29)	Yes	Yes	Yes	Yes	Yes	Yes	Yes	Yes	Yes	Yes	Yes
Kim et al. (30)	Yes	Unclear	Yes	Yes	Yes	Yes	Yes	Yes	Unclear	Yes	Yes
Thijs et al. (31)	Yes	Yes	Yes	Yes	Yes	Yes	Yes	Yes	Yes	Yes	Yes
Atkin et al. (32)	Yes	Unclear	Yes	Yes	Unclear	Yes	Yes	Yes	No	Yes	Yes
Van Hooren et al. (33)	Yes	Yes	Yes	No	Yes	Yes	Yes	Yes	Yes	Yes	Yes

① Was the evidence-based question clearly defined? ② Were the inclusion criteria appropriate? ③ Was the search strategy comprehensive? ④ Were databases/resources sufficiently covered? ⑤ Were quality appraisal tools valid? ⑥ Was independent dual-reviewer screening applied? ⑦ Were data extraction errors minimized? ⑧ Was the study synthesis methodologically sound? ⑨ Was publication bias assessed? ⑩ Were recommendations evidence-driven? ⑪ Were future research needs identified?

#### Expert consensus

All four included expert consensus statements (15–18) received “yes” ratings for all JBI critical appraisal items, indicating high methodological quality (Table 4).

**Table 4:** Quality evaluation results of the expert consensus (*n* = 4).

Incorporate expert consensus	①	②	③	④	⑤	⑥
Chinese PD Consensus Group et al. (15)	Yes	Yes	Yes	Yes	Yes	Yes
Chinese Dysphagia Consensus Group et al. (16)	Yes	Yes	Yes	Yes	Yes	Yes
Cosentino et al. (17)	Yes	Yes	Yes	Yes	Yes	Yes
Schindler et al. (18)	Yes	Yes	Yes	Yes	Yes	Yes

① Was the source of the viewpoint clearly indicated? ② Were the viewpoints derived from influential experts in the field? ③ Were the proposed viewpoints centered on the interests of the relevant population in the study? ④ Were the stated conclusions based on the results of analysis? Was the expression of the viewpoints logical? ⑤ Were other existing literature referenced? ⑥ Were there inconsistencies between the proposed viewpoints and previous literature?

#### Clinical decision-making

A total of 1 clinical decision was included in this study (10). The clarity of the evidence grading was assessed as “no,” while all other items were assessed as “yes.” The overall quality was considered high and it was included.

### Evidence summary and grading

A total of 18 articles were included in this study, from which 53 best-evidence items were extracted across 7 aspects, mainly involving screening, assessment, rehabilitation interventions, nutritional management, airway management, complication management, and outcome evaluation.

The evidence levels and recommendation strengths in this study were determined strictly in accordance with the JBI and FAME frameworks. Taking the “respiratory-swallow coordination” item (No. 24, Level 1b) as an example, the evidence is derived from two high-quality systematic reviews (26, 30) with consistent conclusions, meeting the JBI Level 1b definition of “evidence derived from high-quality systematic reviews.” The recommendation strength was determined through team evaluation and voting based on the FAME framework (Feasibility, Appropriateness, Meaningfulness, Effectiveness), and the supporting references are annotated in Table 5 for reader traceability.

**Table 5:** Summary of Best Evidence for non-pharmacological Management of Dysphagia in Patients with Parkinson’s Disease.

Evidence category		Evidence content	Recommendation level
**Screening**	Screening indications and timing	1. During the initial diagnosis of Parkinson’s Disease (PD) and subsequent follow-ups, clinicians must conduct comprehensive history collection and documentation, along with annual dysphagia screening assessments (at least once a year) (21). Key evaluation domains include: (1) General status: Overall health status and disease progression; (2) Dysphagia-specific manifestations: Changes in eating behavior (e.g., prolonged mealtime), post-swallow residue sensation (pharyngeal adherence or persistent discomfort), subjective symptom localization (patient-reported discomfort patterns); (3) Complication surveillance: Aspiration pneumonia, malnutrition, etc. Multisource validation is mandatory: Direct patient interrogation supplemented by caregiver reports (17, 21).	5a
		2. Swallowing difficulty screening is required for Parkinson’s disease (PD) patients when they exhibit the following symptoms or signs (22): (1) Excessive drooling, weakened tongue movement and strength, prolonged mealtime, and difficulty swallowing tablets; (2) a sensation of food residue remaining in the throat after swallowing, or persistent discomfort; (3) coughing, choking sensation, or an abnormal voice (such as gurgling) during eating or drinking; (4) weight loss or a low body mass index (BMI < 20 kg/m^2^) accompanied by recurrent lower respiratory tract infections.	5a
		3. Screening high-risk populations: (1) PD patients with a daily dose of levodopa of 475 mg or more (17); (2) PD patients who experience early postural instability and gait disturbances (17); (3) Patients with mild cognitive impairment (PD-MCI) (22); 4)PD patients at Hoehn & Yahr stage II or higher (22).	5a
		4. Conducted during the ON (active) phase, when the medication is effective and symptoms are under stable control, to ensure the accuracy of screening (22).	5a
	Screening tools and methods	5. It is recommended to use a dual-track screening model that combines ‘questionnaire/scale assessment’ with ‘swallow test’. For questionnaire screening, prioritize self-report tools such as the Swallowing Disorder Questionnaire (SDQ) or the Munich Dysphagia Test (MDT-PD) (22).	5a
		6. Recommended standardized core screening tools include the Swallowing Disorder Questionnaire (SDQ), Munich Dysphagia Test (MDT-PD), Radboud Oral Motor Phonetic Screening (ROMP), and the Swallowing Clinical Assessment Score (SCAS-PD) (16, 17, 22).	5a
		7. Recommended supplementary screening tools include the Eating Assessment Tool (EAT-10) and the Handheld Cough Test (HCT) (16, 17).	5a
	Managing positive screening results	8. If any screening result is positive, a comprehensive clinical evaluation and diagnostic tests must be conducted immediately to determine the specific details of the dysphagia (16).	5a
		9. It is recommended that a multidimensional, systematic evaluation be conducted by experts in swallowing difficulties, such as speech therapists, to develop personalized intervention plans. The assessment dimensions include: analysis of the patient’s medical history and overall health status; comprehensive examination of oral motor function; observation and evaluation of food and liquid intake during actual eating (27).	2b
**Assessment**	General principles	10. From screening to safety and residue assessment, through severity grading, and quality of life analysis, a closed-loop management system is formed (10, 16, 17, 21, 23, 27, 31).	5a
		11. Utilize PD dysphagia-specific tools as the core, with non-specific tools serving as supplementary, to avoid reliance on a single method (10, 16, 17, 21, 23, 27, 31).	5a
		12. Assess patients individually, taking into account their cognitive status, periods of symptom fluctuation, and medication/dietary needs (10, 16, 17, 21, 23, 27, 31).	5a
	Evaluate projects and tools	13. Recommendations for safety assessment tools and methods to evaluate aspiration risk include: the Penetration-Aspiration Scale (PAS), the Water/Swallow Test, and the Cough Reflex Test (UTC) (16, 21).	5a
		14. Recommended tool for assessing residue (quantifying food retention in the pharynx or larynx): Yale Residue Scale (YRS) (21).	5b
		15. Recommended tools for assessing severity levels, such as the impact of swallowing disorders on eating functions including dietary restrictions, include the Functional Oral Intake Scale (FOIS) and the Dysphagia Outcome and Severity Scale (DOSS) (16).	5b
		16. Recommendations for tools to assess the quality of life related to swallowing disorders (with a focus on the psychological and social impact on patients, while considering their cognitive abilities): Swallowing Quality of Life (Swal-QoL) and Patient-Reported Outcomes Measurement Information System (PROMIS) 39 (17).	5b
		17. Tools and methods for assessing oral motor function and speech, which include detecting issues such as weak tongue muscles and a wet voice indicating aspiration risk, encompass the Range of Motion for the Mouth (ROMP) scale and assessments of speech intensity and pitch (17, 31).	5b
		18. Recommended methods for assessing medication and diet compatibility include the pill swallowing test and the volume-viscosity test (V-VST) (16, 21, 27).	5b
	Operational recommendations	19. Assess the ‘on’ and ‘off’ periods of Parkinson’s disease patients to prevent symptom fluctuations from influencing the results (16).	5a
		20. Blood oxygen measurement is not recommended: A decrease in blood oxygen saturation of more than 3% has a low correlation with aspiration risk and lacks sufficient sensitivity and specificity (21).	5b
		21. For patients with impaired consciousness or who are uncooperative, evaluate using swallowing provocation tests such as cold stimulation (21).	5b
		22. Salivation problem: Quantifying saliva control ability using the Drooling Rating Scale (DRS) (10).	5a
**Rehabilitation intervention**	Biofeedback therapy	23. In visual biofeedback training, patients learn to adjust the sequence of their swallowing movements by observing real-time video images of their swallowing behavior. Study findings indicate that this intervention is associated with improvements in several metrics, including liquid swallowing rate, pre-swallowing time, duration of pre-swallow surface electromyography (sEMG) activation, and scores on the Swallowing Quality of Life (SWAL-QOL) questionnaire (16, 30).	5a
	Breathing-swallowing coordination training	24. Respiratory-swallow coordination training (RSCT) combined with voluntary cough skill training (VCST) constitutes a multimodal therapeutic approach targeting breathing-swallowing coordination. Evidence from two high-quality systematic reviews (26, 30) demonstrates that this combined intervention may improve respiratory-swallow coordination, reduces penetration and aspiration during deglutition, decreases pharyngeal residue in the valleculae and pyriform sinuses, and mitigates overall dysphagia severity, thereby enhancing airway safety, pharyngeal clearance, and global swallowing function.	1b
	Expiratory muscle strength training	25. Expiratory Muscle Strength Training (EMST) constitutes a resistance-based intervention targeting the expiratory musculature (predominantly the abdominal muscles). Evidence^25^indicates that a 4-week to 3-month EMST protocol may help reduce oral spillage, minimizes pharyngeal residue, and mitigates aspiration risk in patients with neurogenic dysphagia, thereby enhancing swallowing safety.	1a
		26. Evidence (26) demonstrated that a comprehensive rehabilitation program integrating speech exercises with orofacial physical training may help improve expiratory pressure, lingual strength, and volitional swallowing awareness.	1a
	Enhanced athletic swallowing program	27. The Intensive Swallowing Program (ISP) constitutes a 4-week comprehensive intervention protocol conducted 4 days per week, integrating speech therapy with expiratory muscle strength training (EMST). Evidence^25^demonstrates that this program effectively enhances lingual strength and improves drinking safety.	1b
	Downward chin strategy	28. During oral intake, patients should consistently employ the chin-down maneuver (characterized by mandibular retraction during deglutition). This technique constitutes an effective compensatory strategy, with evidence from multiple behavioral intervention studies (18, 26, 30)supporting its routine clinical application.	5b
		29. Evidence indicates (30) that chin-down swallowing training performed twice daily (30 min per session) for 4 weeks may effectively improve dysphagia symptoms, quality of life, and psychological well-being.	1b
		30. Patients are advised to employ the chin-down maneuver when consuming liquids of nectar- or honey-thick consistency. Evidence (18, 26) indicates that this compensatory strategy effectively prolongs oral transit time and increases the frequency of tongue pumping movements, thereby reducing the risk of aspiration and the incidence of pneumonia.	5b
	Lee Silverman voice treatment (LSVT)	31. Lee Silverman Voice Treatment (LSVT) is a standardized intensive treatment protocol comprising 4 weekly sessions over 4 weeks. Evidence (30) indicates that LSVT effectively improves speech disorders and swallowing function.	1b
	Therapeutic singing	32. Patients are recommended to undertake an 8-week regimen of various singing exercises specifically targeting phonatory and respiratory musculature. High-quality evidence (30) demonstrates that this intervention effectively improves swallowing function by enhancing respiratory support and laryngeal control.	1b
	Other rehabilitation	33. (1) Jaw movement training: Interventions include Mendelson’s maneuver, the chin-tuck technique, and K-point stimulation. Clinical guideline evidence (21) indicates that these techniques may be employed to improve swallowing function; (2) Forceful Swallowing: Patients are instructed to consciously perform effortful swallows. Clinical guideline evidence (21) suggests that this exercise may enhance lingual strength and improve swallowing physiology; (3) Shaker Exercise Training: This approach aims to strengthen and improve the endurance of the suprahyoid muscles and upper esophageal sphincter. Clinical guideline evidence (21) confirms that achieving these objectives could effectively reduce pharyngeal residue and lowers aspiration risk; (4) Masako’s swallowing training method: This method enhances anterior movement of the posterior pharyngeal wall through tongue fixation (tongue-hold swallow). Clinical guideline evidence (21) notes that this maneuver increases pharyngeal pressure and accelerates bolus propulsion; (5) Chewing gum or sucking on candy: Training is conducted through chewing gum or sucking on hard candy. Expert consensus (15) indicates that this approach facilitates improvement in swallowing function; (6) Voice and swallowing exercises: The regimen includes sustained phonation, forceful articulation, laryngeal range-of-motion exercises, tongue-base to soft-palate contact, sucking on wet gauze, tongue-in-swallow technique, modified supraglottic swallow, gliding vocalization, and tongue rotation. Evidence (18, 26) demonstrates that these exercises improve swallowing reaction initiation time, increase the frequency of multiple swallows, and enhance pharyngeal-related quality of life.	5b
		34. In recent years, traditional swallowing exercises have shown a trend towards skill-based training, and the therapeutic outcomes of the video-assisted group (It constitutes an integrated rehabilitation approach employing video-based technologies, including videofluoroscopic/endoscopic playback and tele-supervision, to facilitate swallowing movement analysis and motor skill acquisition) appear to be superior to those of conventional treatment (15, 18, 21, 26).	5a
**Nutritional management**		35. Swallowing Function Assessment and Dietary Adjustments: (1) Individualized Diet Plan: Adjust the food texture (such as pudding consistency), liquid thickening level, and bolus size based on the results of the swallowing evaluation (22); (2) Prevention of aspiration: a. High-risk patients should prioritize the use of thicker liquids and employ a ≤ 5 mL per push method (22).; b. Provide various thickening agents to enhance patient compliance (21).	5a
		36. Nutritional Risk Management and Monitoring: (1) Risk Identification: Regular monitoring is required to identify risks of malnutrition and dehydration (18, 21); (2) Screening tools: It is recommended to use highly sensitive and specific tools, such as the Mini Nutritional Assessment (MNA), for quick screening. Specialized tools include the Malnutrition Screening Tool (MST), Nutrition Risk Screening (NRS), Geriatric Nutritional Risk Index (GNRI), Subjective Global Assessment (SGA), and Short Nutritional Assessment Questionnaire (SNAQ) (18, 21, 27).	5a
		37. Medication therapy combined with nutrition: (1) Optimal medication administration strategy: Levodopa should be taken 30 min before meals to enhance absorption efficiency (16, 22); (2) Protein intake management: a. It is recommended to redistribute protein intake, consuming 0.8 to 1.0 grams per kilogram of body weight daily (22); b. Distribute meal times appropriately to minimize interference with drug absorption (16, 22).	5a
		38. Special nutritional support methods (18): 1) Indications for tube feeding: a. Consider tube feeding for individuals experiencing significant weight loss or dehydration; b. For short-term needs (less than 4 weeks), prioritize nasogastric feeding, and for long-term needs, opt for percutaneous endoscopic gastrostomy (PEG); c. PEG does not completely eliminate the risk of aspiration; for patients with gastroparesis, consider PEG combined with jejunostomy tube placement (PEG-J); 2) Principles for end-of-life patients: Avoid invasive nutritional support for patients with severe dementia or those at the end of life.	5b
		39. Multidisciplinary collaboration model (18, 27): (1) Team Composition and Responsibilities: a. A multidisciplinary team, comprising nutritionists, speech therapists, and oral health specialists, must collaboratively develop the plan; b. The plan encompasses medication assessment, respiratory therapy, and nutritional intervention; (2) Scope of Intervention: It includes dietary adjustments, the maintenance of oral hygiene, and the resolution of disease-specific issues.	5a
		40. Special strategies and considerations (22): (1) Non-recommended diet types: We clearly do not advocate for low-protein, gluten-free, or strictly plant-based diets; (2) Constipation management: Supplement with probiotics or prebiotic fermented dairy products, and maintain fiber intake.	5a
		41. Considerations for enteral nutrition: Composite intervention: If safe following PEG placement, continue oral intake (functional training) (18).	5b
		42. Principles of medical nutrition therapy: Personalized nutritional plans should consider adherence monitoring, adequate provision of energy and protein, and quality of life assessment (16).	5a
**Airway management**	Tracheostomy/tracheal intubation management	43. Regular assessments by multidisciplinary teams are required to evaluate: swallowing function, oral-pharyngeal secretions, respiratory function, the ability to cough spontaneously, sputum characteristics, and the efficiency of bronchial secretion clearance (21).	5a
		44. Fiber-optic Endoscopic Evaluation of Swallowing (FEES): A key assessment of secretion clearance ability, voluntary swallowing performance, and laryngeal sensitivity (21).	5a
		45. Tube maintenance and monitoring: Regularly check the tube’s position, fit, patency, and granulation tissue formation (21).	5a
		46. Gradual extubation strategy (21): (1) Restore physiological airflow by intermittently deflating the cuff, capping the tube, or using a one-way valve to enhance laryngeal sensitivity; (2) Gradually decrease the internal tube diameter to reduce airway resistance; (3) Criteria for extubation: continuous cuff deflation with capping for 24–48 h, without complications such as aspiration or respiratory distress.	5a
**Management of Complications**		47. Promptly clear secretions from the patient’s oropharynx and trachea to prevent aspiration, which can lead to infection and pneumonia (21).	5a
		48. Monitor the frequency of the patient’s spontaneous swallowing of saliva, regularly perform oral secretion staining (using the modified Evans blue test), and assess the risk of aspiration (21).	5b
**Effect evaluation**		49. The necessity for long-term effect evaluation: The assessment of intervention effectiveness for swallowing disorders in Parkinson’s disease should be extended to at least 6 months or longer post-intervention, with a focus on the sustainability and long-term impact of the intervention (26).	1b
		50. Nutritional and infection indicators recommendations (24, 25): (1) It is recommended to use indicators such as albumin (ALB), hemoglobin (Hb), and prealbumin (PA) to assess treatment effectiveness; (2) It is suggested to combine the incidence of pulmonary infections as an auxiliary indicator for evaluating efficacy.	2b
		51. Recommended objective assessment tools: It is advocated to use standardized tools such as Fiberoptic Endoscopic Evaluation of Swallowing (FEES), Videofluoroscopic Swallowing Study (VFSS), and the Penetration-Aspiration Scale (PAS) for evaluating treatment efficacy (28).	2a
		52. Treatment risks (28): (1) Deep brain stimulation (DBS) may cause a deterioration in motor function, abnormal mental behaviors (such as aggression, hallucinations), and can affect the efficacy of levodopa, as well as other side effects; (2) Repetitive transcranial magnetic stimulation (rTMS) may lead to short-term adverse reactions, including headaches and insomnia.	2a
		53. Comparison of Treatment Efficacy: Stimulation therapies, such as deep brain stimulation (DBS) and repetitive transcranial magnetic stimulation (rTMS), demonstrate significantly better outcomes than behavioral therapy. However, their potential risks and benefits must be carefully considered (24–26, 28).	2a

For detailed information, refer to Table 5.

(Note: In this evidence summary, certain recommendations (specifically those cited as “16” below) are derived from clinical guidelines addressing broad neurogenic dysphagia, with recommendations based on evidence from broader populations with neurological conditions. The authors consider that the core rehabilitation principles and assessment methods therein are applicable to the management of Parkinson’s disease-related dysphagia; therefore, these are incorporated and presented as the best available evidence.)

## Discussion

### Early comprehensive screening and assessment of swallowing disorders in patients with Parkinson’s disease are fundamental prerequisites

Early and precise identification forms the cornerstone of dysphagia management in Parkinson’s disease. This evidence summary indicates that effective management relies not on a single examination, but on a dynamic, multi-source validated workflow. This workflow includes systematic history-taking during initial and follow-up consultations to identify high-risk clinical phenotypes, such as those on high-dose levodopa therapy or with early postural and gait disturbances. Standardized screening should also be performed during the medication “on” state (22). Importantly, positive screening results must lead to comprehensive evaluations by swallowing specialists, such as speech-language pathologists. For screening, it is recommended to prioritize self-report tools, such as the Swallowing Disorder Questionnaire (SDQ) or the Munich Dysphagia Test for Parkinson’s Disease (MDT-PD) (22). These evaluations should incorporate instrumental assessments like FEES or VFSS (16, 27, 34, 35). Successful implementation depends critically on clinicians’ ability to judiciously select and interpret assessment tools. This process must account for disease-specific symptom fluctuations, thereby requiring enhanced professional judgment (17, 21, 23). Consequently, establishing institutional quality assurance and training mechanisms is essential. These safeguards ensure the scientific rigor and continuity of the clinical pathway from screening to intervention.

### Long-term and effective rehabilitation training is an important measure for managing swallowing disorders in Parkinson’s disease

Rehabilitation interventions show potential to improve swallowing function through multiple pathways. Current evidence-based approaches mainly rely on four mechanistic categories.

1. Sensory feedback and motor coordination enhancement: such as visual biofeedback training, which enables patients to modulate swallowing timing through real-time observation of deglutition dynamics (e.g., ultrasonography or laryngoscopic imaging), thereby improving oropharyngeal coordination (16, 30); and respiratory-swallow coordination training, which employs specialized respiratory exercises (e.g., swallowing following forced expiration) to re-establish physiological synchrony between deglutition and respiratory cycles, thereby reducing aspiration risk associated with airway compromise during swallowing (26, 30).
2. Targeted muscular strengthening: such as expiratory muscle strength training (EMST), which augments expiratory muscle groups (e.g., abdominal musculature) through progressive resistance exercises to enhance cough efficacy and post-deglutitive airway clearance (30); and tongue resistance training utilizing pressure sensors or direct resistance loading to specifically augment lingual strength and endurance (21).
3. Behavioral compensatory strategies: such as the chin-down maneuver (chin tuck), which mechanically widens the hypopharyngeal inlet and provides laryngeal coverage by approximating the chin toward the thorax during deglutition, serving as an effective temporary strategy for reducing liquid aspiration (18, 26, 30); and postural adjustments such as head turning and lateral tilting, which exploit gravitational forces to direct the bolus toward the more patent pharyngeal side (21).
4. Innovative applications of conventional techniques: such as video-assisted training (utilizing videofluoroscopic or endoscopic recordings for motor learning), intensive swallowing protocols (high-intensity, structured comprehensive regimens), and adaptation of Lee Silverman Voice Treatment (LSVT) principles (emphasizing high-effort vocalization) to oropharyngeal musculature training, all demonstrating superior efficacy relative to conventional therapeutic approaches (15, 18, 21, 26, 30).

However, research in this field faces some limitations. Most evidence comes from small, short-term trials. These studies lack direct comparisons between different interventions. Therefore, they cannot determine an optimal treatment regimen. Furthermore, follow-up data on long-term efficacy and recurrence risk are still scarce (26, 30). These gaps highlight that current evidence is insufficient to guide individualized, long-term rehabilitation.

Future research should prioritize multicenter, large-scale randomized controlled trials. Such trials are needed to validate the cost-effectiveness of various protocols. Researchers should also explore the development of personalized prescriptions using artificial intelligence. These steps will help advance swallowing rehabilitation toward precision-oriented care.

### Personalized nutritional intervention acts as a complementary strategy to alleviate the nutritional risks associated with swallowing disorders in patients with Parkinson’s disease

Dysphagia and nutritional risk are causally linked. This evidence supports a shift from isolated management to integrated dysphagia-nutrition care (36–38). A core principle is that all dietary modifications, such as fluid thickening or texture alteration, must be based on swallowing function assessments. These modifications should also be coordinated with medication timing, for example with levodopa, to ensure both safety and efficacy (16, 21, 22). Furthermore, routine care should include systematic nutritional risk screening, using tools like the Mini Nutritional Assessment (MNA) (18, 21, 27). For patients needing specialized nutritional support, decisions require careful thought. Indications for enteral feeding, such as percutaneous endoscopic gastrostomy (PEG), must be clear. It is important to recognize that enteral feeding does not fully eliminate aspiration risk. For end-stage patients, overly invasive support should be avoided (18). Multidisciplinary team collaboration is crucial in this process. It ensures comprehensive management, covering needs from dietary design and oral care to constipation management (18, 22, 27).

### Effective airway management is a crucial safeguard to minimize complications associated with swallowing disorders in patients with Parkinson’s disease

For Parkinson’s disease patients with dysphagia complicated by tracheostomy or intubation, meticulous airway management is critical to prevent fatal complications. This evidence summary emphasizes that such management should be based on dynamic assessment by multidisciplinary teams. Comprehensive monitoring of swallowing function, secretion characteristics, and airway clearance capacity is essential (21, 39, 40). Objective tools such as FEES show significant value in evaluating airway protection in this population (35). Core strategies include standardized artificial airway care, tolerance-based extubation/decannulation protocols, and proactive secretion clearance to reduce pneumonia risk (21, 40). Future research could explore integrating intelligent monitoring into this workflow. This may improve the precision and efficiency of clinical decisions (41, 42).

### Balancing potential risks and benefits of interventions

In clinical practice, the potential risks and benefits of each intervention must be carefully weighed. High-intensity rehabilitation can induce muscle fatigue. Over-reliance on thickening agents may impair hydration and reduce medication adherence (22). Invasive procedures such as percutaneous endoscopic gastrostomy (PEG) placement carry surgical and catheter-related risks and may lead to oropharyngeal disuse (18). Moreover, the multidisciplinary team model itself demands considerable healthcare resources and coordination (18, 27). Therefore, shared decision-making is essential. Individualized protocols should be developed based on each patient’s specific condition, preferences, and risk tolerance, with ongoing safety monitoring.

### Considerations for dopaminergic pharmacotherapy in the management of dysphagia in Parkinson’s disease

Although our study focuses on non-pharmacological interventions, the influence of dopaminergic medications—particularly levodopa—on swallowing function should not be overlooked. The ameliorative effects of dopaminergic therapy on dysphagia exhibit inter-individual heterogeneity and stage-specific variability (22, 29, 33). In patients with early-stage disease or those experiencing pronounced “OFF” phase symptoms, levodopa may transiently enhance swallowing efficiency and safety by facilitating the initiation and coordination of oromotor movements (38). Nevertheless, such improvements may not be unstable (38). Furthermore, pharmacologically induced motor fluctuations merit attention, as they may introduce variability in swallowing performance and heighten aspiration risk (38). Dopaminergic pharmacotherapy should be conceptualized as an integral component of comprehensive management strategies in clinical practice (22, 29, 33). Future therapeutic approaches should prioritize dysphagia assessment and rehabilitation during the “medication ON” state, concurrently educating patients and caregivers regarding swallowing hazards associated with the “OFF” phase, to achieve synergistic integration of pharmacological and non-pharmacological interventions.

## Limitations

This study considered only literature in Chinese and English during the search, excluding research in other languages, which may have resulted in missing information. Additionally, the present evidence synthesis focuses primarily on dysphagia management in Parkinson’s disease. While clinical guidelines regarding broad neurogenic dysphagia were consulted to inform assessment and rehabilitation strategies, evidence specifically concerning dysphagia in atypical Parkinsonian syndromes (e.g., multiple system atrophy, progressive supranuclear palsy) was not systematically retrieved. Despite overlapping clinical features and initial management considerations between atypical Parkinsonian syndromes and Parkinson’s disease, fundamental distinctions exist in underlying pathophysiology, disease course, and treatment responsiveness. Consequently, the extrapolation of recommendations from this summary to patients with atypical Parkinsonian syndromes requires careful consideration, with their generalizability remaining to be established in future studies.

## Conclusion

This study has compiled the best available evidence on the management of swallowing disorders in patients with Parkinson’s disease. The evidence spans seven areas, with a total of 53 best evidence items identified. The overall quality of the evidence is high and can provide reference suggestions for related clinical practice. However, when applying these suggestions, it is necessary to consider local national conditions, cultural background, and lifestyle habits to develop personalized management plans for patients with Parkinson’s disease who experience swallowing disorders. In the future, high-quality original studies in this area could be conducted to provide a scientific foundation for enhancing the relevant management strategies.

## Data Availability

The original contributions presented in the study are included in the article/supplementary material, further inquiries can be directed to the corresponding author/s.
